# The surface modification and characterization of SiO_2_ nanoparticles for higher foam stability

**DOI:** 10.1038/s41598-020-76464-w

**Published:** 2020-11-10

**Authors:** Mansoo Choi, Wang-Kyu Choi, Chong-Hun Jung, Seon-Byeong Kim

**Affiliations:** Decommissioning Technology Research Division, Korea Atomic Energy Research Institute, Daejeon, 305-353 Korea

**Keywords:** Engineering, Materials science

## Abstract

The surfactant and colloidal nanoparticles has been considered for various applications because of interaction of both complex mixtures. The hydrophilic SiO_2_ nanoparticle could not be surface active behavior at the liquid/air interface. In this study, the SiO_2_ nanoparticles have been modified with 3-isocyanatopropyltriethoxy-silane (ICP), and the effect of foam stability has been investigated. The physical properties of surface modified SiO_2_ nanoparticle were analyzed by XRD, TGA, FT-IR, and SEM. After surface modification of SiO_2_ nanoparticles, the contact angle of SiO_2_ nanoparticle was also increased from 62° to 82° with increased ICP concentration. The experimental result has shown that SiO_2_ nanoparticle with ICP was positive effect and improved foam stability could be obtained at proper ICP concentration compared with un-modified SiO_2_ nanoparticle.

## Introduction

The surfactant and nanoparticles has been considered for various applications because of interaction of both complex mixtures, which could influence the dynamic and static behavior^1–4^. Silica (SiO_2_) nanoparticles have been widely used in various applications such as catalyst, biomolecule separations, chromatographic supports^5,6^. SiO_2_ nanoparticles are also used as foam stabilizers for the foams and emulsion applications^7,8^. Generally, aqueous foams are thermodynamically unstable because of breaking of the film or irreversible drainage of the liquid. It is reported that colloidal hydrophobic particles are able to significantly improve the foam stability^8^. These approaches are being used for the stable foam applications. However, as synthesized mesoporous silica nanoparticles have hydrophilic properties, which is not adsorb and surface active behavior at the liquid/air interface. Therefore, the use of hydrophilic SiO_2_ nanoparticles has been disadvantageous. In order to enhance the foam stability, the surface modification of SiO_2_ nanoparticle should be needed. Normally, the modified SiO_2_ nanoparticles could stabilized the foam by interfacial elasticity^9^. A number of methods surface modification of SiO_2_ nanoparticles have been developed by using organic or in inorganic additives^10–12^. The SiO_2_ nanoparticle surface has been changed from hydrophilic to hydrophobic properties, resulting from incorporation with functional groups. It is reported that the hydrophobic SiO_2_ nanoparticle has enhanced affinity to organic compound^10^.

In this study, the surface modification of SiO_2_ nanoparticles has been prepared by copolymerization with organic-silica precursors in the presence of a cetyltrimethylammonium bromide. Our approach has shown in a one-pot synthesis method and did not add any co-solvent or additives during the synthesis of SiO_2_ nanoparticles. After modification of the SiO_2_ nanoparticle, the morphology and structure were analyzed by SEM and XRD. Foam stability has been improved with SiO_2_ nanoparticles modified by 3-isocyanatopropyltriethoxy-silane.

## Result and discussion

**Figure 1:**
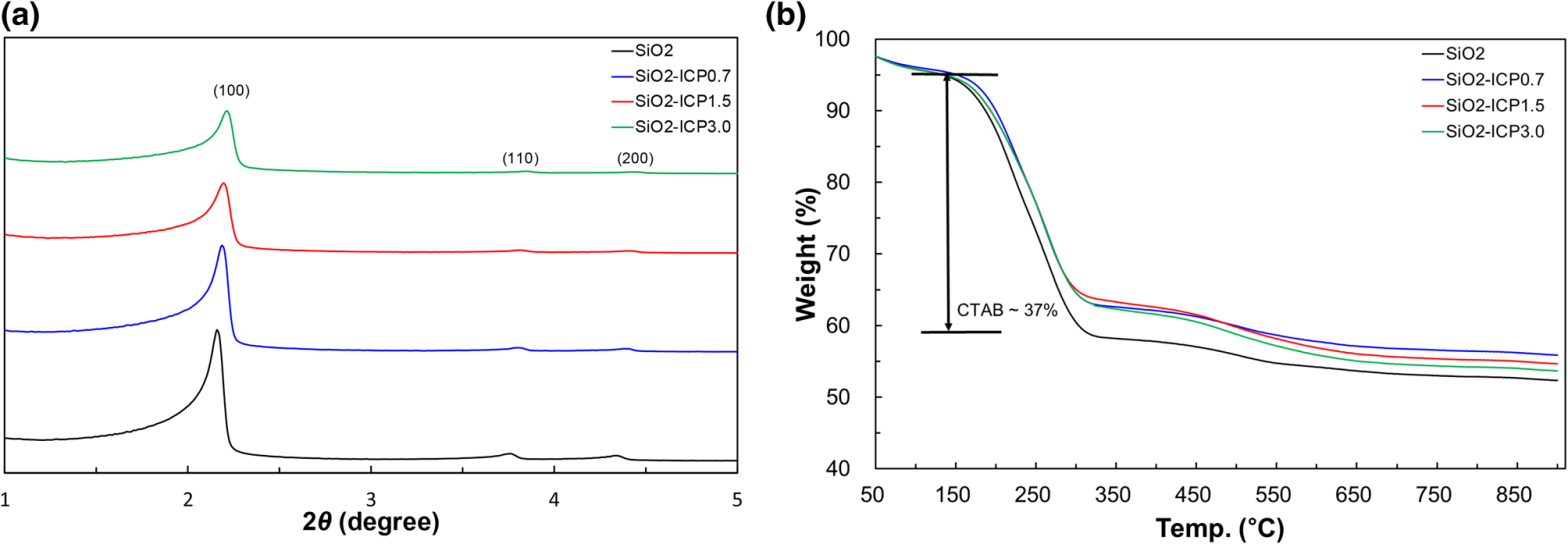
The low-angle X-ray diffraction patterns and TGA thermograms of SiO_2_ nanoparticles as a function of the ICP concentration.

Figure 1a shows the small-angle XRD patterns of SiO_2_ and SiO_2_-ICP samples synthesized according to the ICP concentration. All SiO_2_-ICP samples show an intense (100) peak and two weak (110, 200) peaks, indicating that the presence of three peaks is common in SiO_2_ materials and 2D hexagonally ordered structure. The intensity of *d*(100) in the XRD peaks were decreased as a function of the ICP concentration. It is indicating that the addition of ICP did not influence the SiO_2_ structure, but resulted in well-ordered structure when ICP was added during the synthesis^13^. Moreover, the addition of ICP resulted in the decrease of SiO_2_ nanoparticle size, but not in the collapse in the SiO_2_ mesoporous structure^13^. The TGA profiles of as-synthesized SiO_2_ particles are depicted in Fig. 1b. The first region from room temperature to 150 °C is attributed to the release of absorbed water on the silica surface. The drastic weight loss (37%) caused by decomposition of organic molecule and CTAB in the SiO_2_ matrix was observed in the temperature range of 150–300 °C. The weight loss after 550 °C was decreased, indicating that the pure SiO_2_ particles can be obtained. And further weight loss was observed after 500 °C according to the ICP concentration, which is decomposed of ICP modification.

**Figure 2:**
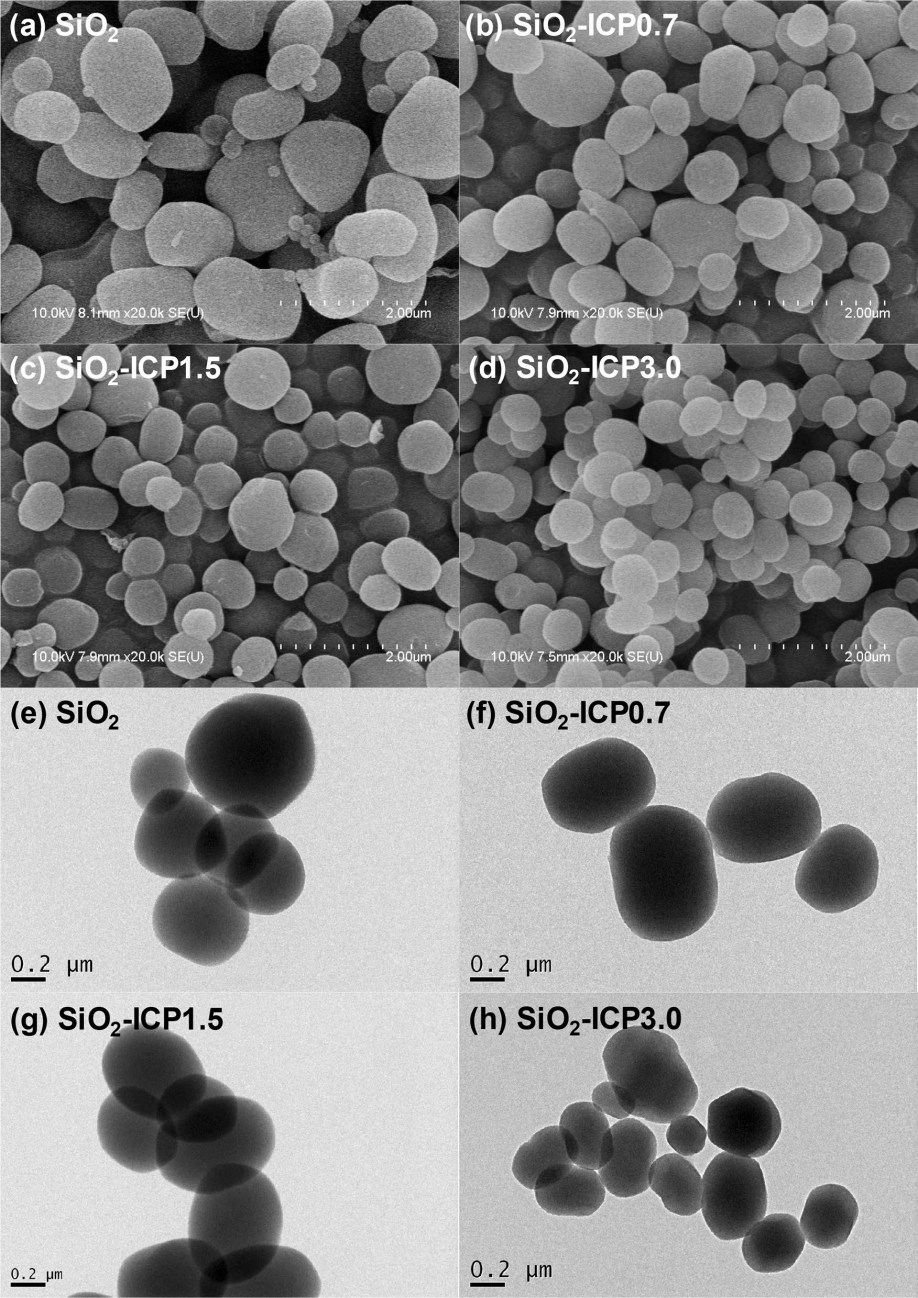
SEM and TEM images of SiO_2_ nanoparticles according to the ICP concentration.

The morphology of SiO_2_ and SiO_2_-ICP particles was observed by FE-SEM and TEM. Figure 2 shows the SEM and TEM images of pristine SiO_2_ and SiO_2_-ICP particles. As shown in Fig. 2, SEM and TEM image is clearly reveal that the morphology of SiO_2_-ICP nanoparticles was transformed from kidney-bean-shape to spherical shape according to the ICP concentration. It also observed that the particle size of SiO_2_ decreased with higher ICP concentration (500 nm < Fig. 2d,h). Moreover, the mesopore structures of SiO_2_ are also observed in spite of their different particle size (Fig. 2e–h). From the XRD, SEM, and TEM, the addition of ICP could modify the morphology and size of SiO_2_ particles during SiO_2_ synthesis.

**Figure 3:**
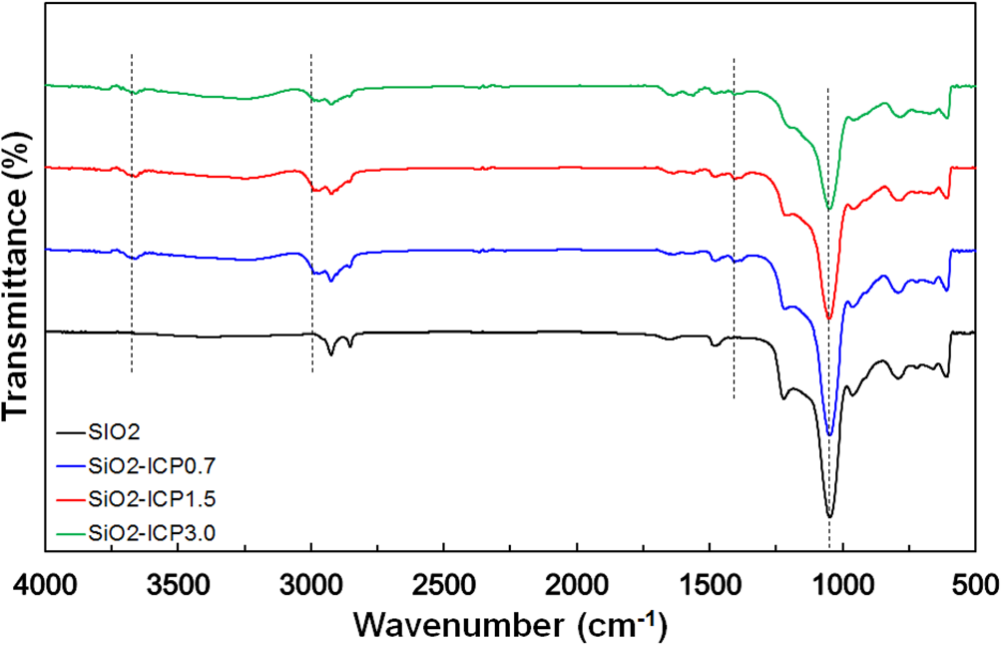
FT-IR spectra of SiO_2_ nanoparticles as a function of the ICP concentration.

The surface modification of SiO_2_ particles was investigated by FT-IR. Figure 3 shows the FT-IR spectra of SiO_2_ and SiO_2_-ICP samples. The peak at 1060 cm^−1^ was attributed to the Si–O–C asymmetry stretching vibration and Si–OH stretching vibration^14^. After modification of ICP, the new peaks were observed at 2974 and 3700 cm^−1^, which is assigned to C–H stretching vibration in CH_2_ and CH_3_ and the 1387 cm^−1^ peak was corresponding to the vibration of C–H, which was not observed in SiO_2_ particles but were in SiO_2_-ICP particles^15^. This is the fact that the ICP was effectively grafted on the SiO_2_ surface. The intensity peak of at 1060 cm^−1^ was decreased as a function of ICP concentration, indicating the surface modification of SiO_2_ particles by ICP.

**Figure 4:**
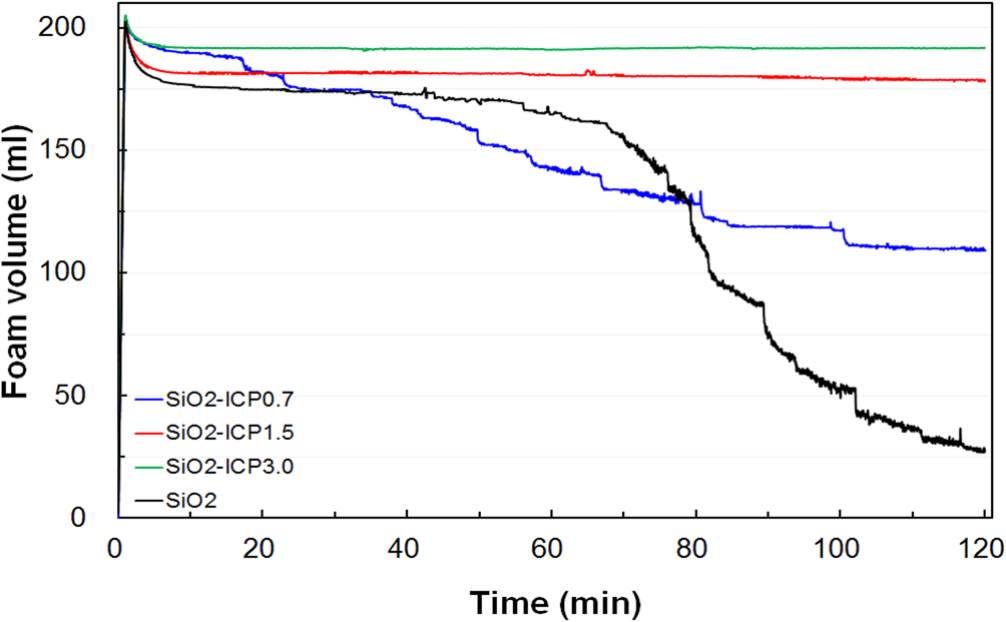
Foam volume measurement of SiO_2_ nanoparticles according to the ICP concentration.

For the foam stability measurement, the aqueous foam was generated and its stability was recorded by using Foamscan analyzer. The N_2_ gas was used to produce the foam volume of 200 ml. After that, the gas flow was stopped and foam volume was monitored according to time. Figure 4 shows the decay of the foam volume of surfactant which contained 1 wt% SiO_2_ particles (EM 100, 1 wt%) at pH 2. All the samples show the similar foam volume properties and the foam stability of SiO_2_-ICP particles and exhibit higher foam stability than SiO_2_ particles without ICP. The foam stability was improved with increasing ICP concentration. It is considered that the hydrophobic particles can be attached on the liquid–gas interfaces and stabilize the foam bubbles in surfactant-free diluted suspensions^16–19^. Moreover, it is reported that the smaller particle leads to higher viscosity of the suspension under identical particle geometry and type^20^. Therefore, the high ICP concentration could be favor the foam stability because of modification of wettability of SiO_2_ particles at the air–water interface and aqueous solution.

**Figure 5:**
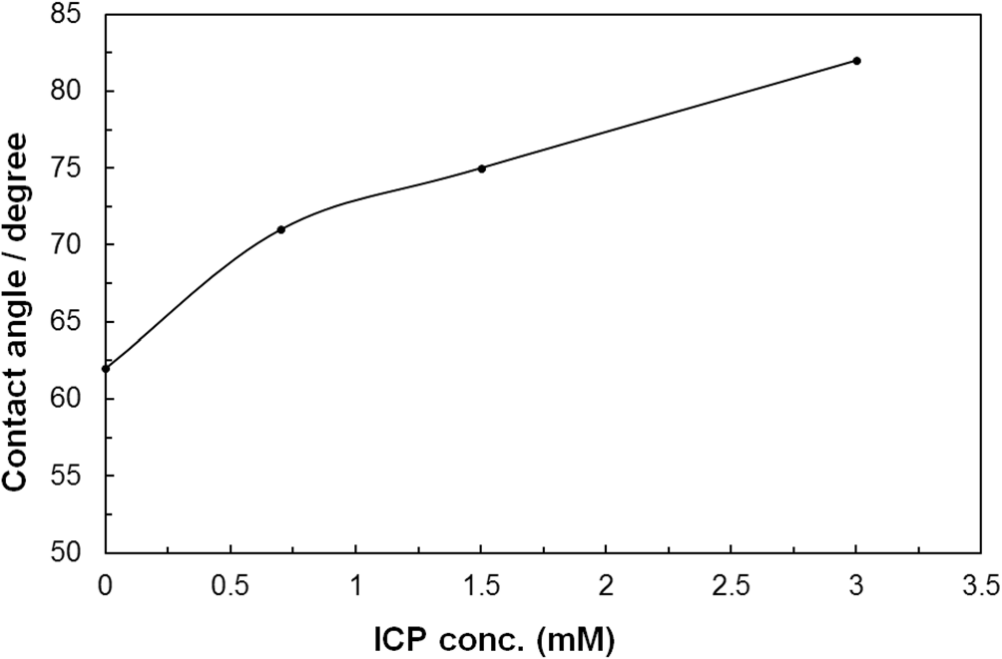
Contact angles of SiO_2_ nanoparticles as a function of the ICP concentration.

In order to clarify the improvement of foam stability of SiO_2_ nanoparticles, we have evaluated the contact angles of SiO_2_-ICP samples in air. The particle-stabilized silica nanoparticle depends on significantly on the particle hydrophobicity at the water–air interface in terms of the contact angle^21^. It is reported that the hydrophilicity/hydrophobicity have been determined by contact angle method, which is important evidence for affecting properties of the particle surface^22^. Figure 5 shows the contact angle of SiO_2_ nanoparticles with increasing ICP concentration. As shown in Fig. 5, in the absence of ICP, the contact angle is 62°. However, the ICP concentration is increased, contact angle was also increased from 62° to 82°. The result of contact angle of SiO_2_-ICP nanoparticle matched well with the foam stabilities. The SiO_2_ nanoparticle surface thus undergo transition more hydrophobic wettability, indicating that more ICP molecules are adsorbed on the SiO_2_ nanoparticle surface. It can be concluded that the organic groups of ICP affect the surface properties in the SiO_2_ nanoparticles.

## Conclusion

In this study, we synthesized SiO_2_ nanoparticles through sol–gel process and modified its surface by adjusting ICP concentration. The surface modification of SiO_2_ nanoparticles was analyzed by XRD, TGA, SEM, TEM, FT-IR and contact angle. The morphology of silica nanoparticle transformed from kidney-bean-shape to spheres according to the ICP concentration and contact angle was increased as the ICP amount was increased, which became more hydrophobic. The results of TGA and FT-IR have shown the interaction between the SiO_2_ nanoparticle surface and ICP after the surface modification. The foam stability was gradually increased with increasing ICP amount, which means that the hydrophobic properties of SiO_2_ nanoparticle affect the foam stability and foam stabilization.

## Methods

The SiO_2_ nanoparticles were synthesized by following a published procedure^23^. Typically, the cetyltrimethylammonium bromide (CTAB, 2.0 g) and 2 M sodium hydroxide solution (NaOH, 7.0 ml) were added in deionized water and the mixture was stirred and heated at 80 °C. To clear this solution, tetraethylorthosilicate (TEOS, 9.3 ml) and desired 3-isocyanatopropyltriethoxy-silane (ICP) were added to the solution via rapid injection. The withe precipitation was observed after 3 min and the solution was maintained at 80 °C for 2 h. After the reaction time, the as-synthesized particles were washed by water and methanol, and dried under vacuum oven.

The morphology of SiO_2_ nanoparticles were analyzed by a field emission scanning electron microscopy (FE-SEM, Hitachi), a high resolution transmission electron microscope (HR-TEM, JEOL). Small angle X-ray diffraction (XRD, PANalytical) analysis was conducted by using Cu Kα radiation (λ = 1.5405) in a range of 0.5°–5° 2*θ*. Thermogravimetric analysis (TGA, Mettler-Toledo) was carried out to measure the concentration of CTAB in SiO_2_ nanoparticles under flowing N_2_ with a heating rate of 5 °C min^−1^. The fourier transform infrared spectroscopy (FT-IR) spectra of SiO_2_ nanoparticles was recorded in FT-IR spectrophotometer (VERTEX 80). Contact angle of water drops on SiO_2_ nanoparticles was determined using Phoenix series.

The foam stability was analyzed with commercially available Foamscan instrument (Teclis/IT Concept). Foamscan is commercially available instrument to measure foamability, foam stability, and foam drainage^24^. For the foam stability, Elotant™ Miloside 100 (EM 100, LG Household and Health Care) was used as surfactant and as-synthesized SiO_2_ nanoparticles (1 wt%) were added to the surfactant solution to investigate the effect of foam stability which contains as-synthesized SiO_2_ nanoparticles according to the ICP concentration.
